# Impact of obesity on breast cancer recurrence by menopausal status and subtype: a retrospective cohort study

**DOI:** 10.1007/s10549-025-07823-2

**Published:** 2025-10-21

**Authors:** K.-H. Yoon, Y. Yoon, S. Jeong, J. Kang, J. H. Oh, H. W. Koh, H.-C. Shin, E.-K. Kim

**Affiliations:** Department of Surgery, Seoul National University Bundang Hospital, Seoul National University College of Medicine, 82 Gumi-ro 173 Beon-gil, Bundang-gu, Seongnam-Si, Republic of Korea 13620

**Keywords:** Breast cancer, Body mass index, Menopause, Histological subtype

## Abstract

**Purpose:**

To evaluate the effect of body mass index (BMI) on oncologic outcomes in patients with breast cancer stratified by menopausal status and histological subtype. Although studies have focused on the relationship between obesity and breast cancer risk, the association between BMI and breast cancer recurrence after surgery remains controversial.

**Methods:**

This retrospective study included patients who underwent curative surgery for breast cancer between June 2003 and November 2017. Normal weight and overweight groups were defined based on the World Health Organization classification. The primary outcome was recurrence-free survival, evaluated at 1, 5, and 10 years after curative surgery. Patients were stratified by BMI category, histological subtype, and menopausal status. The main measures included tumor characteristics, recurrence events, and survival outcomes across groups.

**Results:**

Among 4506 patients included in the analysis, 3384 (75.1%) had luminal-type breast cancer. The overweight group (n = 1259) was associated with older age (normal weight (NW): 50.2 ±10.9 vs. overweight (OW): 56.5 ± 1.9, *P* < 0.001) and higher T stage (≥ T2: NW: 1226 (37.7%) vs. OW: 577 (45.8%), *P* < 0.001). In patients with luminal-type breast cancer, 10-year recurrence-free survival was significantly worse in the overweight group (NW 89.3% vs. OW 85.7%, *P* = 0.018). Subgroup analysis showed that premenopausal patients with luminal-type breast cancer who were overweight had an increased risk of recurrence (*P* = 0.003).

**Conclusions:**

Obesity is a significant, potentially modifiable risk factor for recurrence in premenopausal females with luminal-type breast cancer.

## Introduction

Being overweight or obese is an increasing global health burden [1]. Obesity is a major risk factor for major illnesses, including cardiovascular disease, diabetes, and cancer [2–4]. Increasing evidence shows that patients who are obese are not only at higher risk of malignancy but also experience higher rates of recurrence and cancer-related death after diagnosis [5–7]. In breast cancer, a recent meta-analysis encompassing 82 studies found an association between body weight increase and increased breast cancer mortality [8]. Obesity and other modifiable lifestyle factors, including alcohol consumption and smoking, have also been linked to higher contralateral breast cancer development risk after primary breast cancer diagnosis [9].

Although obesity is generally associated with worse survival outcomes in patients with breast cancer, breast cancer shows heterogeneity, with diverse molecular subtypes characterized by differential expression profiles of selective markers [10–12]. Previous research on breast cancer risk has found conflicting results regarding the association of outcomes with obesity according to menopausal status [13, 14]. A higher body mass index (BMI) also differentially impacts the risk of breast cancer subtypes [15–17].

Although most studies have focused on the role of obesity in breast cancer development and the association between body weight and all-cause mortality in patients with breast cancer, the effect of BMI on breast cancer recurrence has been examined less. Subgroup analyses encompassing different subtypes and menopausal statuses are scarce, and no definite conclusions have been drawn. This study aimed to evaluate the effect of BMI on oncological outcomes in patients with breast cancer who underwent curative surgery, stratified by histological subtype and menopausal status.

## Methods

### Study design

A total of 4650 patients who underwent curative surgery for pathologically confirmed breast cancer between June 2003 and November 2017 at Seoul National University Bundang Hospital (SNUBH) were initially considered. Patients diagnosed with stage IV disease at diagnosis (n = 4), with synchronous bilateral breast cancer (n = 88), or with a history of surgery for contralateral breast cancer (n =18) were excluded. Because menopausal status was a crucial factor in the analysis, male patients (n = 21) and those missing menopausal status data (n =13) were also excluded. Ultimately, 4506 patients were included in the final analysis. This study was approved by the Institutional Review Board of SNUBH (No. B-2001-586-117) and was prepared in compliance with the Strengthening the Reporting of Observational Studies in Epidemiology (STROBE) guidelines for cohort studies [18].

### Data collection and definitions

Clinical characteristics, including anthropometric measurements, menopausal status, family history, operative data, and pathological information of the study participants, were obtained from medical records. The pathological information included tumor size, TNM stage, estrogen receptor (ER) status, progesterone receptor (PR) status, human epidermal growth factor receptor-2 (HER2) expression, and Ki-67 index. Molecular subtypes were defined based on ER, PR, and HER2 expression levels using the St. Gallen expert consensus panel [19]. Luminal A and B subtypes were combined into one group (luminal) and compared with HER2 and triple-negative (TNBC) subtypes.

Patients were classified into normal weight(NW) and overweight(OW) groups based on the World Health Organization(WHO) criteria [20]. The WHO criteria for obesity defines BMI < 18.5 kg/m^2^ as underweight, 18.5–24.9 kg/m^2^ as normal weight, 25–29.9 kg/m^2^ as overweight, and > 30 kg/m^2^ as obese. In the total cohort, 179 patients (4.0%) were underweight, 3068 patients (68.1%) were normal weight, 1061 patients (23.5%) were overweight, and 198 patients (4.4%) were obese. Since the proportions of patients in the underweight and obese groups were small, we dichotomized the patients into the NW and OW groups using a BMI cutoff of 25 kg/m^2^.

Postoperative administration of chemotherapy, hormone therapy, or radiotherapy, and details of recurrence were extracted from the same records. Recurrences included local, regional, and systemic conditions. Local recurrence was defined as recurrence in the ipsilateral breast, including the nipple-areolar complex and skin flap. Regional recurrence was defined as recurrence in the ipsilateral axillary lymph nodes. Systemic recurrence encompassed all types of distant metastases, including but not limited to the liver, lung, bone, and brain. Contralateral breast cancer was excluded as a recurrence because it could not be clearly differentiated from de novo malignancy. Follow-up details were recorded until the most recent hospital visit or the date of death for each patient.

### Statistical analysis

All categorical data are expressed as the frequency (percentage), and continuous variables are expressed as the mean ± standard deviation for normally distributed variables or as the median (interquartile range) for non-normally distributed variables. Continuous variables were compared using the paired *t*-test or Mann–Whitney U test according to the distribution of data. Categorical variables were compared using Pearson’s *χ*^2^ test or Fisher’s exact test. Survival analyses were conducted using the Kaplan–Meier method and log-rank test. All *P*-values were two-sided, with *P* < 0.05 considered statistically significant. Statistical analyses were performed using R (version 4.5.0).

## Results

### Baseline characteristics

The mean age of the study participants was 50.2 years in the NW group and 56.5 years in the OW group (*P* < 0.001; Table 1). The proportion of postmenopausal females was significantly higher in the OW group (NW, 1236 (38.1%) vs. OW, 779 (61.9%); *P* < 0.001). The NW and OW groups did not differ significantly in family history of breast cancer (354 (10.9%) vs. 136 (10.8%), respectively; *P* = 0.965), breast cancer susceptibility gene (*BRCA*) mutations (71 (2.2%) vs. 20 (1.6%), *P* = 0.241), or neoadjuvant chemotherapy administration (457 (14.1%) vs. 186 (14.8%), *P* = 0.579).

**Table 1:** Baseline characteristics of normal weight (NW) and overweight (OW) groups

	NW (*n* = 3247)	OW (n = 1259)	*P*-value
Age(mean), years	50.2 ± 10.9	56.5 ± 11.9	< 0.001
Sex, female	3247 (100%)	1259 (100%)	
BMI(mean), kg/m^2^	21.8 ± 12.2	27.7 ± 16.9	
Menopause			< 0.001
Premenopausal	2011 (61.9%)	480 (38.1%)	
Postmenopausal	1236 (38.1%)	779 (61.9%)	
Family history of breast cancer	354 (10.9%)	136 (10.8%)	0.965
Number of affected family members			0.465
0	2894 (89.1%)	1123 (89.2%)	
1	325 (10.0%)	119 (9.5%)	
2	22 (0.7%)	14 (1.1%)	
3	6 (0.2%)	3 (0.2%)	
BRCA mutation	71 (2.2%)	20 (1.6%)	0.241
BRCA1	31 (1.0%)	12 (1.0%)	
BRCA2	41 (1.3%)	8 (0.6%)	
Neoadjuvant chemotherapy	457 (14.1%)	186 (14.8%)	0.579

*BMI* body mass index, *BRCA* breast cancer susceptibility gene

### Operative parameters and pathological features

The operative and pathological features of the patients are shown in Table 2. Axillary lymph node dissection was performed more often in the OW group than the NW group (1046 (32.2%) vs. 477 (37.9%), respectively; *P* = 0.001). The OW group had a larger tumor size, higher T stage, and higher N stage. The groups did not differ significantly in hormone receptor expression, HER2 expression, or Ki-67 index. The luminal subtype was dominant in both groups, followed by TNBC and HER2-positive subtypes.

**Table 2:** Operative parameters and pathological features of normal weight (NW) and overweight (OW) groups

	NW (*n* = 3247)	OW (n = 1259)	*P*-value
Operation type			0.061
Breast-conserving surgery	1916 (59.0)	782 (62.1)	
Total mastectomy	1331 (41.0)	477 (37.9)	
Axillary approach			0.001
None	27 (0.8)	8 (0.6)	
Sentinel lymph node biopsy	2174 (67.0)	774 (61.5)	
Axillary lymph node dissection	1046 (32.2)	477 (37.9)	
T stage			< 0.001
(y)pT0	14 (0.4)	7 (0.6)	
(y)pT1	2246 (69.2)	760 (60.4)	
(y)pT2	893 (27.5)	448 (35.6)	
(y)pT3	68 (2.1)	34 (2.7)	
(y)pT4	26 (0.8)	10 (0.8)	
Lymph node metastasis	1130 (34.8)	475 (37.7)	0.071
N stage			0.019
N0	2224 (68.5)	817 (64.9)	
N1	735 (22.6)	307 (24.4)	
N2	167 (5.1)	91 (7.2)	
N3	121 (3.7)	44 (3.5)	
Grade			0.698
1	2015 (62.1)	798 (63.4)	
2	1113 (34.3)	415 (33.0)	
3	119 (3.7)	46 (3.7)	
Hormone receptor expression			
ER	2434 (75.0)	951 (75.5)	0.717
PR	2111 (65.0)	798 (63.4)	0.321
Histological subtype			0.706
Luminal A/B	2434 (75.2)	950 (75.8)	
Triple negative	461 (14.2)	182 (14.5)	
HER2	342 (10.6)	122 (9.7)	

*ER* estrogen, *PR* progesterone, *HER2* human epidermal growth factor receptor 2

### Association between BMI and breast cancer recurrence

**Fig. 1:**
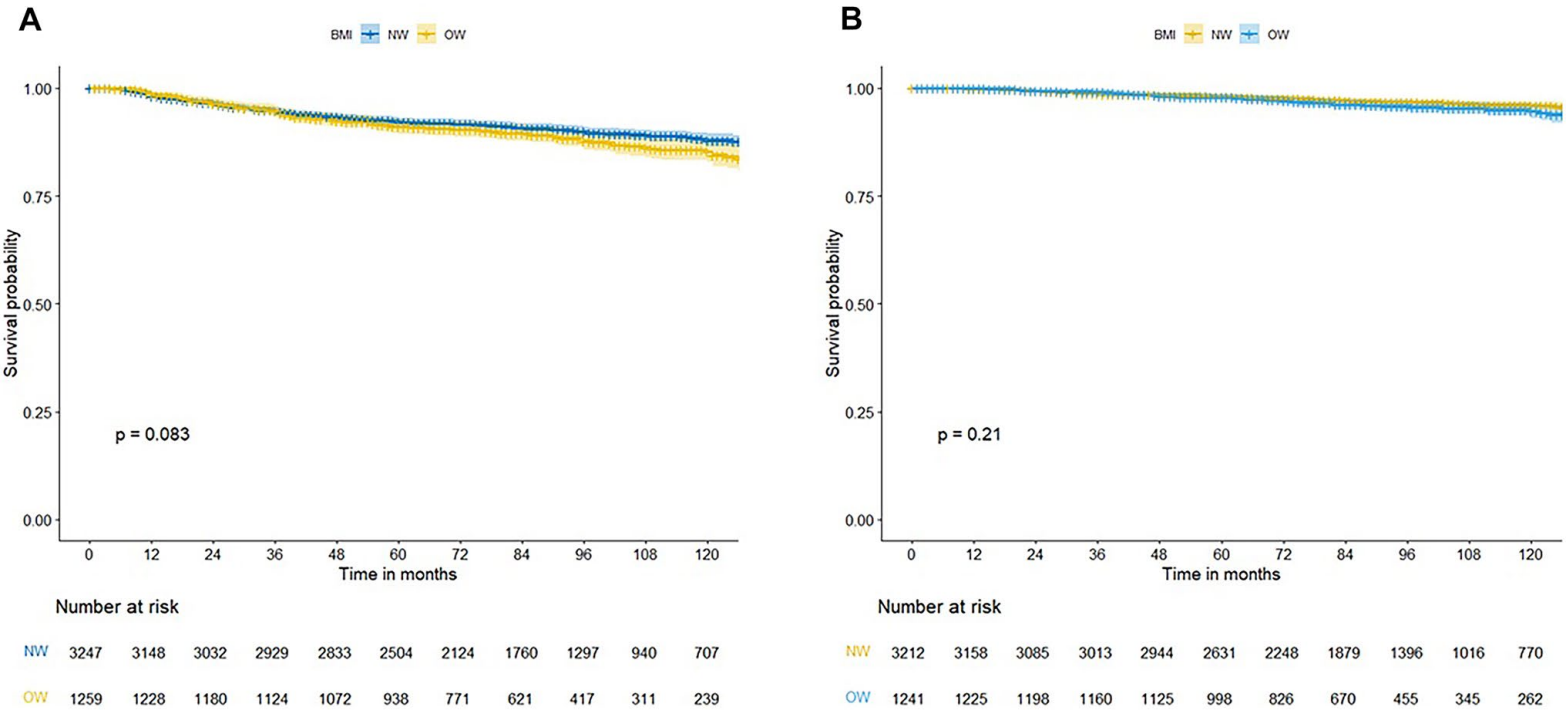
Comparison of survival outcomes between normal weight (NW) and overweight (OW) groups. **a** Recurrence-free survival **b** Overall survival

The two groups showed no difference in adjuvant treatment rates, including hormone therapy, chemotherapy, and radiotherapy (Table 3). The NW group was associated with a higher rate of tamoxifen therapy (67.9%), while tamoxifen and aromatase inhibitor prescription rates were similar in the OW group (44.7% and 40.8%, respectively). Chemotherapy adherence rate was 98.0% (2010/2050) and 97.5% (777/797) in the NW and OW groups, respectively (*P* = 0.644). During a median follow-up period of 88 months, recurrence was noted in 491 patients. 1 year, 5 year and 10 year RFS in the NW and OW groups were 98.2%, 92.2%, and 88.0%, and 98.7%, 91.1%, and 85.3%, respectively (*P* = 0.083; Fig. 1a). Although overall recurrence rates showed no statistically significant differences, the distant metastasis rate was higher in the OW group (249 (7.7%) vs. 121 (9.6%), *P* = 0.038). Breast cancer-related deaths were 3.4% (109/3,213) in the NW group and 4.2% (52/1,243) in the OW group (*P* = 0.260). 1 year, 5 year, and 10 year OS in the NW and OW groups were 99.9%, 98.1%, and 94.2%, and 100%, 97.9%, and 93.0%, respectively (*P* = 0.210; Fig. 1b).

**Table 3:** Adjuvant treatment and survival outcomes in normal weight (NW) and overweight (OW) groups

	NW(*n* = 3247)	OW(n = 1259)	*P*-value
Adjuvant treatment			
Hormone therapy	2377 (73.2)	919 (73.0)	0.915
Tamoxifen	1613 (67.9)	411 (44.7)	
Aromatase inhibitor	542 (22.8)	375 (40.8)	
Mixed	222 (9.3)	133 (14.5)	
Chemotherapy	2050 (63.1)	797 (63.3)	0.943
Chemotherapy adherence	2010 (98.0)	777 (97.5)	0.644
Radiotherapy	2313 (71.2)	915 (72.7)	0.354
Follow-up duration (mean), months	94.1±41.1	90.8±40.7	0.014
Recurrence	340 (10.5)	151 (12.0)	0.156
Local	101 (3.1)	47 (3.7)	0.338
Regional	102 (3.1)	39 (3.1)	>0.999
Distant	249 (7.7)	121 (9.6)	0.038
Death			0.173
Cancer-related	109 (3.4)	52 (4.1)	
Other cause	43 (1.3)	21 (1.7)	
Unknown	2 (0.1)	3 (0.2)

### Association between BMI and breast cancer recurrence by subtype and menopausal status

**Fig. 2:**
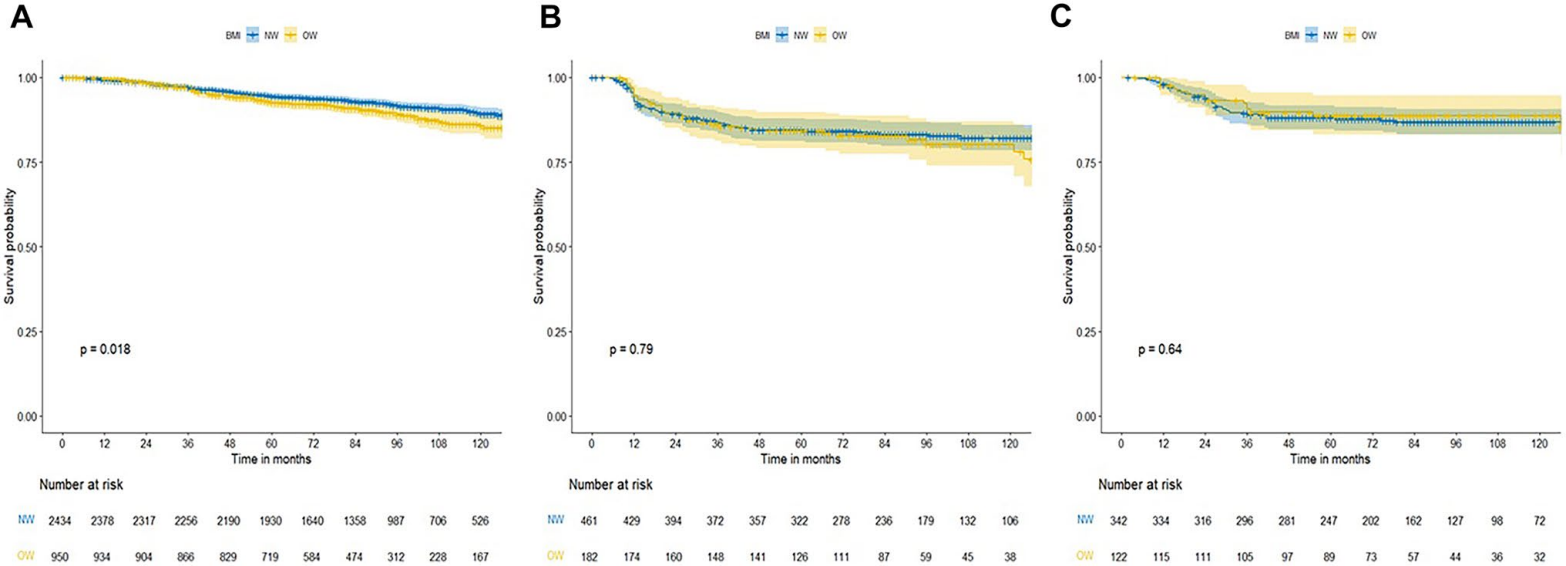
Comparison of survival outcomes between normal weight (NW) and overweight (OW) groups according to histological subtype. **a** Luminal subtype, **b** triple negative subtype, **c** HER2 subtype

Subgroup analysis was performed to compare RFS between the NW and OW groups according to cancer subtype. For the luminal subtype, 5-year RFS was significantly worse in the OW group (NW: 89.3% vs. OW: 85.7%; *P* = 0.018; Fig. 2a). Five-year RFS rates were not significantly different between groups for the TNBC (NW: 82.1% vs. OW: 80.2%, *P* = 0.790; Fig. 2b) or HER2 subtypes (NW: 86.8% vs. OW: 88.7%, *P* = 0.640; Fig. 2c).

**Fig. 3:**
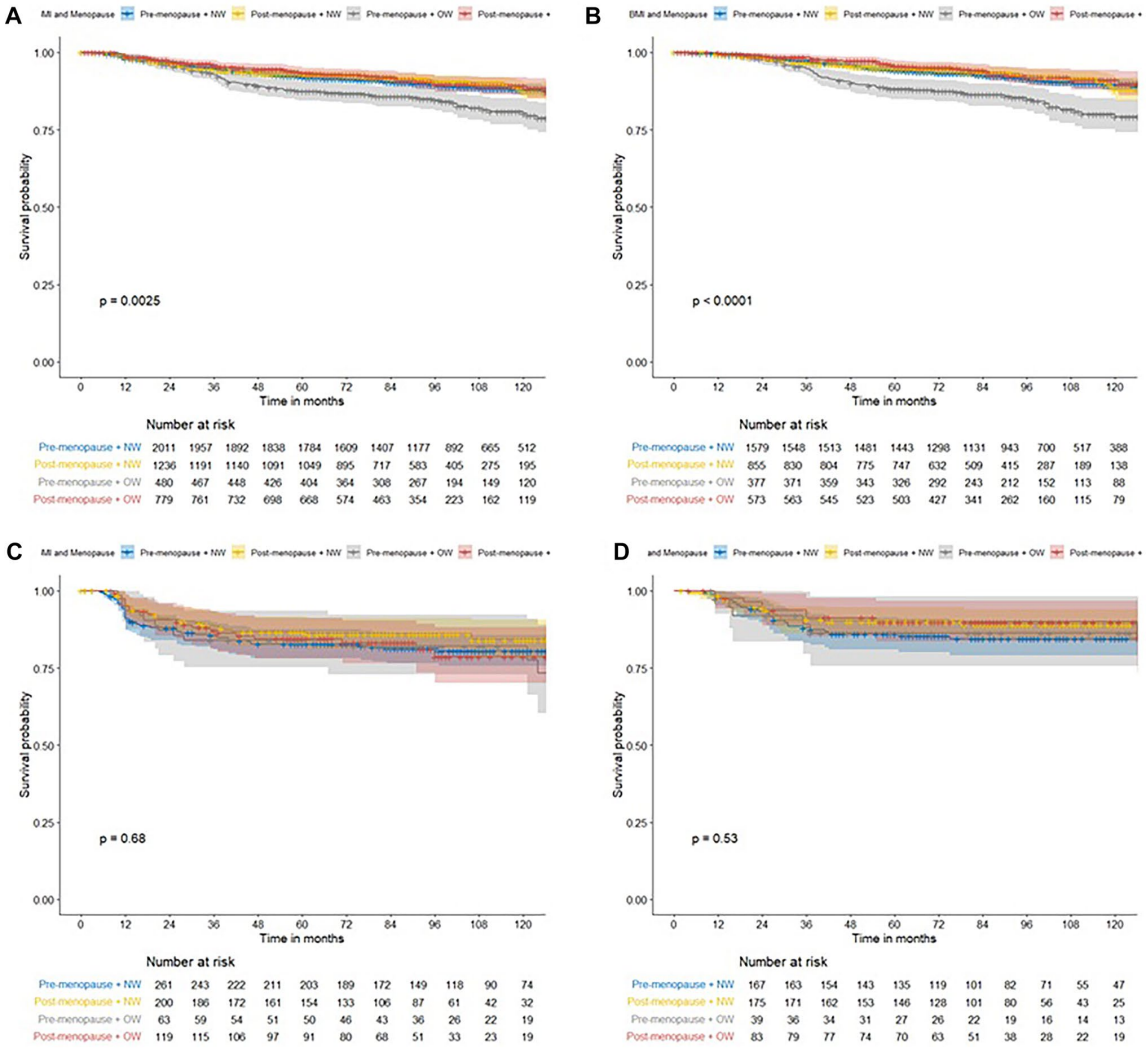
Comparison of survival outcomes between normal weight (NW) and overweight (OW) groups according to menopausal status and histological subtype. **a **Total cohort, **b** Luminal subtype, **c** triple negative subtype, **d** HER2 subtype

BMI and menopausal status were combined to form four subgroups. Premenopausal patients in the OW group had worse survival outcomes compared to all other subgroups (premenopausal NW: 88.0%, postmenopausal NW: 87.5%, premenopausal OW: 80.3%, postmenopausal OW: 89.1%; *P* = 0.003; Fig. 3). This difference was seen only in the luminal subtype (premenopausal NW: 89.6%, postmenopausal NW: 88.0%, premenopausal OW: 79.2%, postmenopausal OW: 91.1%; *P* < 0.001). In the TNBC and HER2-positive subtypes, survival outcomes were not affected by the combination of BMI and menopausal status.

## Discussion

This study evaluated the association between BMI and breast cancer prognosis in Korean females. Higher BMI was associated with more aggressive tumor characteristics, as reported in previous studies [21–23]. Although overall recurrence and mortality rates were not significantly different between the NW and OW groups, the distant metastasis rate was higher in patients in the OW group. The subgroup analysis revealed an interplay between hormone receptor expression, menopausal status, and BMI. For the luminal subtype of breast cancer, poorer RFS was observed in the OW group. When menopausal status and BMI measurements were combined, premenopausal patients in the OW group had the worst survival outcomes; this association persisted only in the luminal subtype.

The association between BMI and breast cancer prognosis has been actively investigated [3, 8]. Ewertz et al. found that obesity was associated with older age and more advanced disease in a Danish cohort; the risk of distant metastasis after 10 years and the risk of breast cancer mortality after 30 years were significantly increased in patients with BMI > 30 kg/m^2^ [24]. A recent meta-analysis also found that patients with breast cancer who were overweight or obese had poorer OS than those of normal weight [25]. However, results regarding which specific subgroups of patients with breast cancer would be most affected by BMI are conflicting. Several studies have reported that the therapeutic benefit of hormone therapy decreases with increasing BMI, suggesting that patients with hormone receptor-positive breast cancer who are obese are at a higher risk of recurrence and mortality [26–28]. Aromatase inhibitors are less effective in patients who are obese because of the high expression level of aromatase in peripheral fat. Some studies have also found that obesity is a poor prognostic factor in hormone receptor-positive breast cancer in premenopausal patients [29]. In contrast, results from the International Breast Cancer Study Group trials suggested that premenopausal patients receiving chemotherapy without endocrine therapy were especially affected by BMI [30]. The complexity of evidence suggests that further evaluation of the relationship between BMI and breast cancer prognosis considering both tumor biology and hormonal status is necessary.

An important point of consideration is ethnic differences in the association between obesity and breast cancer. While several studies have argued that BMI and breast cancer risk show an inverse relationship in premenopausal females, systematic reviews have also found that this relationship was only statistically significant in certain ethnic groups [13, 31, 32]. Amadou et al. concluded that each 5 kg m^2^ increase in BMI was associated with a 7% and 5% reduction in risk in Caucasian and African females, respectively; however, in Asian females, a 5% increase in risk was observed [32]. These differences could be a result of different body compositions across ethnic groups. In general, Asians are more likely to have higher levels of overall abdominal body fat compared to Caucasians for the same given BMI [33]. Obesity-related metabolic diseases also develop at a lower BMI in Asian patients [34]. Reflecting such differences, the Asia–Pacific criteria sets a lower BMI cut-off for overweight and obesity [35]. Many large-scale studies on the association between body weight and breast cancer survival have been conducted in non-Hispanic white women, and evidence in other ethnic groups remain scarce [36]. As this study evaluated exclusively Korean females, the poorer prognosis in premenopausal patients in the OW group could be partly explained by such ethnic differences as well. Additionally, previous studies based on Western cohorts included a substantial number of obese (BMI > 30 kg/m^2^) patients and analyzed them separately [24, 26–28]. On the contrary, only 4.4% of the study population was obese in the current study, and the mean BMI of the OW group was 27.7 kg/m^2^. This was consistent with previous studies reporting a much lower BMI and Asian and Asian American groups compared to other ethnicities [36–38]. Although future large-scale studies are necessary, the results of the current study suggest that survival in Asian women with breast cancer may be impacted to a greater degree by excess adiposity, especially in premenopausal patients with hormone receptor positive tumors.

Breast cancer is highly influenced by hormones such as estrogen and progesterone, and several hormone-related risk factors such as parity, age at menarche, childbearing history, and breastfeeding have been explored [39–41]. Early onset breast cancer, typically defined as breast cancer diagnosed in patients younger than 40 years, is gradually increasing; an important trend is the increase in other modifiable factors, including physical inactivity, western-style diet, sugar-sweetened beverage intake, and concomitant obesity in this population [42–44]. Most early-onset breast cancer patients are presumed to be premenopausal considering the age span, and the results of the current study suggest that obesity is a modifiable risk factor in this patient population. Guidelines for breast cancer survivors suggest that a healthy lifestyle, including physical activity and dietary changes, could lead to improved quality of life and decreased death from breast cancer or any cause [45, 46]. For patients who are overweight or obese before surgery, postoperative weight reduction of 5–10% is recommended for improved survival outcomes [47]. However, there is no clear algorithm targeting specific patient subgroups with detailed intervention protocols and clear goals. To date, no prior study has specifically evaluated the impact of lifestyle interventions on oncological outcomes in specific subgroups of breast cancer patients. Future prospective studies evaluating the differential effect of physical activity and dietary changes in patients with different tumor subtypes, body composition, and hormonal status could broaden the clinical implications of our findings. Increased weight management therapy has been associated with significant weight loss and a lower risk of cardiovascular events in previous studies [48, 49]. Several randomized controlled trials are in progress to evaluate the effect of weight loss interventions on survival outcomes in early-stage breast cancer [49–51]. These studies could further shed light on whether active weight reduction during adjuvant treatment could improve survival outcomes in premenopausal patients with luminal subtype breast cancer.

This study has certain limitations. First, it was limited by its retrospective nature with potential selection bias. Second, overweight NW and OW patients were categorized based solely on BMI. Although BMI is the most commonly used measure of overweight and obesity, it does not full reflect the patient’s body composition. Studies including other measurements of body composition such as waist circumference, waist:hip ratio, and quantitative body compartment measurements based on computed tomography or magnetic resonance imaging could predict the association between obesity and breast cancer recurrence more accurately [52]. Future studies should incorporate multimodal assessment of body composition to properly evaluate the association between obesity and breast cancer survival. Lastly, as the study population was recruited at a single tertiary referral center in Korea, ethnical differences and potential interactions with obesity could not be evaluated.

In conclusion, this study evaluated the association between BMI and breast cancer prognosis according to breast cancer subtype and menopausal status in a large cohort of Korean females. Our results suggest that premenopausal patients with breast cancer who are overweight at diagnosis may benefit from active weight reduction and lifestyle modifications during adjuvant treatment and long-term follow-up.

## Data Availability

The data that support the findings of this study are available on request from the corresponding author, E.-K. Kim. The data are not publicly available due to information that could compromise the privacy of research participants.
